# The Application of High-Dose Proton Pump Inhibitor Induction Treatment before Dual Therapy for *Helicobacter pylori* Eradication: An Open-Label Random Trial

**DOI:** 10.3390/jcm10194352

**Published:** 2021-09-24

**Authors:** Li-Wei Chen, Liang-Che Chang, Chung-Ching Hua, Ching-Jung Liu, Tien-Shin Chou, Chih-Lang Lin, Rong-Nan Chien

**Affiliations:** 1 Department of Gastroenterology and Hepatology, Chang-Gung Memorial Hospital and University, Keelung Branch, Keelung 20401, Taiwan; cjliuu@cgmh.org.tw (C.-J.L.); f139859@gmail.com (T.-S.C.); lion@cgmh.org.tw (C.-L.L.); ronald@cgmh.org.tw (R.-N.C.); 2 Community Medicine Research Center, Chang-Gung Memorial Hospital and University, Keelung Branch, Keelung 20401, Taiwan; 3 Department of Pathology, Chang-Gung Memorial Hospital and University, Keelung Branch, Keelung 20401, Taiwan; lc2008@cgmh.org.tw; 4 Department of Internal Medicine, Chang-Gung Memorial Hospital and University, Keelung Branch, Keelung 20401, Taiwan; hc2008@cgmh.org.tw

**Keywords:** *Helicobacter pylori*, proton pump inhibitor, rabeprazole, amoxicillin, drug therapy, combination

## Abstract

This was a prospective, randomized, open-label trial. Patients without previous *Helicobacter pylori* eradication therapy were randomly assigned to either a high-dose dual therapy (HDDT) group or a traditional clarithromycin/amoxicillin triple therapy (CATT) group. In the HDDT group, patients took rabeprazole, 20 mg, four times per day for three days and then dual therapy with rabeprazole, 20 mg, and amoxicillin, 500 mg, four times per day during the patient’s breakfast, lunch, dinner, and bedtime for 14 days. In the CATT group, patients received conventional triple therapy for 14 days (rabeprazole 20 mg, amoxicillin 1 g, and clarithromycin 500 mg twice per day). In the HDDT group, the success rates of *H. pylori* eradication were 91.7% (95% confidence interval (CI): 0.78–0.97) by intention-to-treat (ITT) and 94.3% (95% CI: 0.79–0.99) by per-protocol (PP) analysis. In the CATT group, the eradication rates were 77.1% (95% CI: 0.61–0.87) by ITT and 84.3% (95% CI: 0.66–0.94) by PP analysis. The study completion rates were 97.2% (35/36) in the HDDT group. Three-day high-dose rabeprazole induction treatment before dual therapy and a schedule of taking the drug at meal and bed times could achieve an acceptable *H. pylori* eradication rate (>90%) and good drug compliance.

## 1. Introduction

The *Helicobacter pylori* infection is common worldwide and is strongly associated with gastrointestinal diseases, including peptic ulcers, atrophic gastritis, and gastric cancer [1]. Clarithromycin-based triple therapy is recommended in many guidelines as the first-line therapy for the treatment of *H. pylori* infection. Because of the increasing antibiotic resistance of *H. pylori*, the eradication rate of clarithromycin-based triple therapy has decreased below 80% [2,3]. Other combinations for eradication therapy, such as sequential therapy, concomitant therapy, bismuth quadruple therapy, and levofloxacin-based triple therapy, have all been recommended as first-line or rescue therapies [2,3]. The eradication rates of these combinations have been reported to be below 90% by intention-to-treat (ITT) analysis [4]. Clarithromycin resistance cannot be overcome by increasing the dose and duration of treatment [3,5]. However, *H. pylori* resistance to amoxicillin is rare [6,7]. Amoxicillin can be applied for *H. pylori* eradication therapy by increasing the dose or frequency to increase the eradication rate [6,7]. Although antimicrobial susceptibility testing is recommended in regions of high antibiotic resistance, it is technique-dependent and not readily available in most areas [2,3]. Therefore, amoxicillin can be used empirically without the need for susceptibility culture testing [6,7]. 

The bactericidal effect of amoxicillin against *H. pylori* is time- and pH-dependent [8,9,10]. Amoxicillin is more stable at a higher intragastric pH (>5.5), which can be achieved by intake of high-dose proton pump inhibitors (PPIs) [10,11,12,13]. High-dose PPIs for *H. pylori* eradication also has a direct effect on antimicrobial activity [14]. *H. pylori* infections are thought to enter a non-replicative but viable state when the pH in their microenvironment is within 3 to 6 [12]. The bactericidal effect of amoxicillin depends on bacterial replication. When organisms are in this non-replicative but viable state, they are phenotypically resistant to amoxicillin [15]. The site harboring the phenotypically resistant organism is most likely within the mucus layer, based directly on the measurement of *H. pylori* gene expression within the stomach [13]. Increasing the pH in this layer to between 6 and 7 theoretically would allow the bacteria to enter the replicative state where they would become susceptible to amoxicillin [15]. To achieve the best condition for eradicating *H. pylori*, it is reasonable to increase the intragastric pH using high-dose PPI before eradication therapy.

Dual therapy by combining amoxicillin and PPI twice daily did not achieve a satisfactory cure rate in the past [16]. Subsequent studies found that the effectiveness of dual therapy could be improved by administering both PPI and amoxicillin at a high dose and frequency [17,18]. High-frequency (every six hours per day) and -dose (3 g per day) amoxicillin usage could maintain a steady plasma concentration above the minimum inhibitory concentration to maintain the bactericidal effect against *H. pylori* [8,9,19]. However, drug adherence should be confirmed in the situation of four times and 14 days [19]. A fixed time schedule, such as a six-hour interval, would allow patients to take the drugs in the early morning or at midnight. Moreover, the standard prescription was to take PPIs before meals and amoxicillin after meals since patients must take PPIs and amoxicillin separately. To elevate the intragastric pH, increasing the frequency of PPI intake before dual eradication therapy should be done. 

The hypotheses of the current study were as follows:

1. Three-day high-dose rabeprazole (20 mg, four times per day) induction treatment before *H. pylori* eradication would increase intragastric pH and induce active replication in *H. pylori*. The active replicative status enhances the bactericidal effects of amoxicillin.
2. After a 3-day high-dose rabeprazole induction treatment, rabeprazole and amoxicillin could be taken simultaneously. It is not necessary to separate rabeprazole (before meals) and amoxicillin (after meals). Simultaneous administration of rabeprazole and amoxicillin would improve drug compliance.
3. A total of 2 g instead of 3 g amoxicillin (500 mg, four times per day) might be adequate for dual therapy to achieve an acceptable eradication rate.
4. A modified schedule such as taking the medications during breakfast, lunch, dinner, and bedtime could adopt the patients’ daily activities. This schedule might remind patients to take drugs and achieve good drug compliance.

This study aimed to evaluate whether high-dose rabeprazole induction treatment before dual eradication therapy and taking 14-day rabeprazole and amoxicillin simultaneously at meal and bed times could achieve an acceptable *H. pylori* eradication rate and drug compliance.

## 2. Materials and Methods

This was a prospective, randomized, open-label trial. From January 2019 to July 2021, a community-based study, screening for *H. pylori* infection using the C^13^ urea breath test (UBT) was performed in the northeastern region of Taiwan. Patients with UBT-confirmed *H. pylori* infection were referred to the Keelung Chang-Gung Memorial Hospital. Esophagogastroduodenoscopy (EGD) was performed for all patients who had symptoms such as epigastric pain, black stool passage, or body weight loss. The exclusion criteria were a history of *H. pylori* eradication therapy, allergy to the study drug (rabeprazole, amoxicillin, and clarithromycin), antibiotic treatment within 1 month before entering this study, taking PPI or H_2_ blockers within 14 days, age < 18 years, and current pregnancy.

### 2.1. UBT

^13^C-UBT was performed after an overnight fast using the Proto Pylori kit (Isodiagnostika, Montreal, Quebec, Canada) containing 75 mg of ^13^C-urea and additives. Two breath samples obtained within a 30-min interval were analyzed using gas chromatography/isotope ratio mass spectrometry. The results were expressed as delta over baseline (DOB). A local validation test with a DOB cut-off value of 3.5 yields a sensitivity of 96% (95% confidence interval (CI): 93–99%) and a specificity of 98% (95% CI: 93–100%) relative to the manufacturer’s reference.

### 2.2. Treatment Regimens

Patients were randomly assigned to two groups for *H. pylori* eradication therapy using the number sequence on a random table. The investigator marked a number one by one on a random table when a new participant entered the trial. Participants who received an odd number were assigned to the high-dose dual therapy (HDDT) group. In the HDDT group, oral rabeprazole (20 mg, four times per day) was administered for 3 days. Then, combined rabeprazole, 20 mg, and amoxicillin, 500 mg, four times per day, were administered as a high-frequency dual therapy for 14 days. The four times per day were scheduled for breakfast, lunch, dinner, and bedtime. Rabeprazole and amoxicillin were taken after meals for 30 min simultaneously.

Patients who received an even number were assigned to the clarithromycin-amoxicillin triple therapy (CATT) group. In the CATT group, 14-day triple therapy (rabeprazole 20 mg, amoxicillin 1 g, and clarithromycin 500 mg twice per day) was administered. Rabeprazole was administered 30 min before meals, and clarithromycin/amoxicillin was administered 30 min after a meal.

During the treatment period, all patients were instructed to avoid acidic foods (e.g., citrus fruits or juices). This was done to minimize the impact of ingested foods on increasing intragastric acidity, which could alter drug activity. Smoking was prohibited during the study period. Subjects were required to record daily drug consumption during the treatment period on a diary card. After completing the treatment course, drug compliance and adverse effects of the treatments were surveyed at outpatient clinics. The incidence of side effects was checked using a standardized degree of interference with daily activities as follows: absent, mild—not interfering, moderate—frequently interfering but allowing treatment to be completed, and severe—requiring interruption of treatment [20].

*H. pylori* eradication success was determined by a negative ^13^C-UBT result four weeks after eradication therapy.

The Institutional Review Board of Chang-Gung Memorial Hospital approved this study (IRB No. 201801792A3C501). All participants agreed to the study conditions and provided informed consent before enrollment in the study. The study was registered at ClinicalTrial.gov (ID: NCT03802318).

### 2.3. Statistical Analysis

The *H. pylori* eradication rate of traditional triple therapy (CATT) is 70–80% as per previous reports [21,22]. The null hypothesis in the current study was that the HDDT group would show an eradication rate as 95% and that the CATT group would show an eradication rate of 70% [21]. The minimum number of patients required was 31 in both groups to obtain a power of 0.8 at a significance level of 0.05. We estimated that the number of patients enrolled was 35 in both groups, with a dropout rate of 10%, to obtain a minimal number of 70 subjects in this study. Continuous data are expressed as mean ± standard deviation. A two-sample t-test was used to compare the mean values. Categorical data were analyzed using chi-square and Fisher exact tests. Both intention-to-treat (ITT) and per-protocol (PP) analyses were performed. All statistical tests were two-tailed. Differences were considered statistically significant at *p* < 0.05. 

An acceptable eradication rate was defined as a PP treatment success of 90% or greater; a treatment success rate of 80% or less was deemed unacceptable. 

Statistical analyses were performed using the Statistical Package for the Social Sciences version 16.0, for Windows (SPSS, Chicago, IL, USA).

## 3. Results

**Figure 1:**
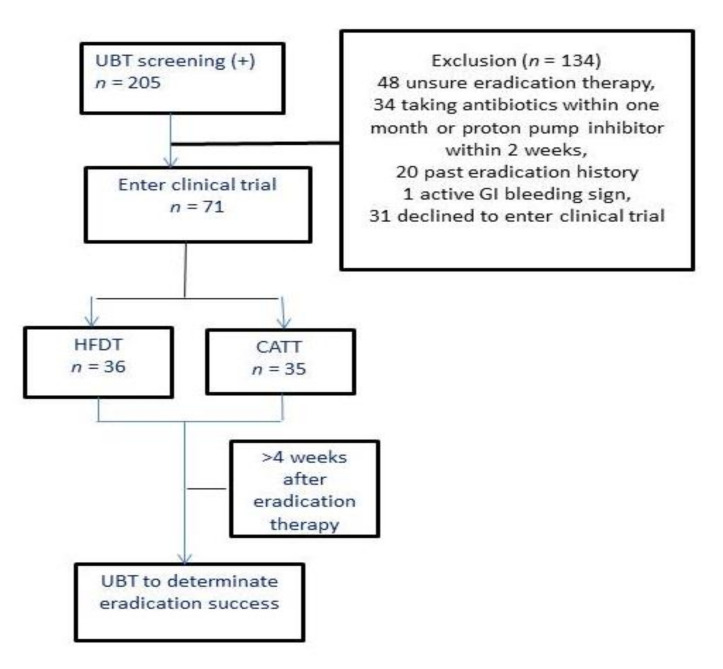
Study diagram.

A total of 205 patients with positive UBT results were enrolled in the study. After the medication history review and clinical evaluation, 134 subjects were excluded. Among these 134 subjects, 48 subjects were unsure of the medical history of *H. pylori* eradication, 20 subjects had a history of *H. pylori* eradication, 34 subjects had taken antibiotics within one month or PPI within two weeks, 1 subject had signs of active gastrointestinal bleeding on admission, and 31 subjects declined to participate in this clinical trial. Seventy-one subjects who had not previously undergone *H. pylori* eradication therapy were randomly assigned to the HDDT group (*n* = 36) and the CATT group (*n* = 35) using a random table (Figure 1). 

There was no statistical difference between the factors of age, sex distribution, smoking/alcohol consumption, endoscopic findings, and clinical presentation. In the HDDT group, the success rate of *H. pylori* eradication was 91.7% (33/36, 95% confidence interval (CI): 78–97%) by ITT and 94.3% (33/35, 95% CI: 79–99%) by PP analysis. In the CATT group, the eradication rates were 77.1% (27/35, 95% CI: 61–87%) by ITT and 84.3% (27/32, 95% CI: 66–94%) by PP analysis (Table 1). The study completion rates were 97.2% (35/36) in the HDDT group and 91.4% (32/35) in the CATT group. One subject in the HDDT group withdrew because of the side effects of headache and constipation during the PPI induction time. Three subjects in the CATT group withdrew because of the side effects of taste distortion (bitter mouth; *n* = 2) and diarrhea (*n* = 1). Drug compliance was good in all groups (>90% complete rate). There was no statistical difference between the *H. pylori* eradication rate and the side effects of the HDDT and CATT groups (Table 2).

The unit prices of amoxicillin (500 mg), rabeprazole (20 mg), and clarithromycin (500 mg) are NTD 4, 11, and 16, respectively. The gross prices of HDDT regimens and CATT regimens are NTD 972 (about EUR 29.77 or USD 35.03) and NTD 980 (about EUR 30.01 or USD 35.32).

## 4. Discussion

Because of the increasing clarithromycin resistance of *H. pylori*, the eradication rate of clarithromycin-based triple therapy is now suboptimal and has an unacceptable efficacy in some Southeastern Asiatic countries [2,3,23]. Although antimicrobial susceptibility testing is recommended for the choice of eradication regimens, it is not available in some areas. A treatment design combining PPI with low-resistance drugs, such as amoxicillin, is more practical without the need for susceptibility testing [19].

Maintaining a high intragastric pH and a steady plasma amoxicillin concentration are important for the success of dual therapy [8,9,10,11,12,13,14]. High-dose PPI induction treatment could achieve a high intragastric pH before dual therapy. Taking amoxicillin four times per day, every six hours, could achieve a steady concentration of amoxicillin [19,21]. However, it is a challenge to suggest that our patients take drugs at midnight or in the early morning when taking drugs every six hours is a requirement in real-world practice. To improve drug compliance, incorporating the drug intake schedule into the patient’s daily activities is important. For most patients, drug intake during meal times (breakfast, lunch, and dinner) and bedtime may be appropriate. Incorporating drug intake in the patient’s daily activities makes it easier to remind them to take their medications regularly. The drawback is the variable interval between bedtime and awakening time. Moreover, the few patients who take only two meals (or less) every day are not suitable for this schedule. The concentration of the drug may fluctuate during this period, and the influence of eradication success should be evaluated. Another concern is the sequence of taking PPI and amoxicillin. To maintain the effect of blocking acid secretion by the gastric proton pump receptor, PPI is suggested before meals. Although the issue of taking amoxicillin pre- versus post-prandial time was debated before, amoxicillin is better taken after meals when the intragastric pH rises. This can maintain the drug’s antibacterial effect [10,11,12,13,21,24,25]. Thus, patients should consider taking PPI before meals and amoxicillin after meals. However, this decreases drug compliance during treatment. To improve drug compliance, taking both PPI and amoxicillin at the same time, such as at the postprandial time, may be a solution. In the current study, a three-day high-dose rabeprazole induction treatment was initiated followed by simultaneous amoxicillin/rabeprazole intake at meal and bed times. During this treatment course, treatment compliance was good, and the antibacterial effect of amoxicillin was not compromised based on the eradication rate (>90%).

According to a study by Yang et al. in Taiwan, combined rabeprazole (20 mg) and amoxicillin (750 mg), taken every six hours, achieved an *H. pylori* eradication rate of 95.3% by ITT and 96.6% by PP [19]. In another dual therapy by Yang et al. from China, combined treatment of esomeprazole (20 mg) and amoxicillin (750 mg) achieved eradication rates of 87.9% and 91.1% by ITT and PP analyses, respectively. The modified dual therapy was administered four times a day (three meals and bed time). Esomeprazole was administered 30 min before meals, rabeprazole was administered 30 min after meals, and both were taken simultaneously one hour before bed time [25]. According to one meta-analysis, a high intragastric pH, a higher dose of amoxicillin, and the duration of treatment were the key factors for successful eradication in dual therapy [26]. The potency of different PPIs to inhibit gastric acid secretion was variable and was also influenced by the CYP2C19 genotype [27]. To overcome the varying potency of PPIs while avoiding interference from CYP2C19, the dosage of PPI may be increased [19,25]. The other solution involves using a potassium-competitive acid blocker, P-CAB, such as vonoprazan [28]. One recent study used dual therapy with vonoprazan (20 mg) and amoxicillin (750 mg) taken twice per day for seven days. This treatment achieved an eradication rate of 84.5% by ITT and 87.1% by PP analysis [28].

The dose interval of amoxicillin, such as 1 g three times a day or 750 mg four times a day, has not yet been unified [28]. Other factors that can affect intragastric pH and antisecretory drug effectiveness include smoking and acidic foods. Yang et al. reported that avoiding acidic foods during treatment was important for eradication success [19]. In our study, a three-day high-frequency rabeprazole induction treatment was used to increase intragastric pH before amoxicillin therapy. The dose of amoxicillin used was 2 g (500 mg four times per day or 1 g twice per day). The success rates of *H. pylori* eradication in the HDDT group were 91.7% and 94.3% by ITT and PP analyses, respectively. The eradication rates using 2 g amoxicillin daily were similar to the results obtained using 3 g amoxicillin daily (more than 90%) [19,25]. All subjects can take the drug four times daily at meal and bed times, which was consistent with the findings from the modified dual therapy of Yang et al. [25].

The limitation of this study was the small number of subjects. The source of the subjects was a community UBT screening for *H. pylori* infection. Because the COVID-19 pandemic developed and removing face masks was forbidden, UBT screening was conducted for a long period in this study. Although the minimal number of subjects was calculated at the start of the study, the statistical power was not strong when only a minimal number of subjects were included. A wide range of 95% CI in the results must be considered with caution. Although the *H. pylori* eradication rates were 91.7% (ITT) and 94.3% (PP) in the HDDT group in this study, there was no statistical difference compared with the eradication rates of 77.1% (ITT) and 84.3% (PP) in the CATT group. One reason for the lack of statistical difference was beta error (type II error) due to the small number of cases. The other reason was more than the predicted eradication rate (70%) achieved in the CATT group (77.1% by ITT and 84.3% by PP analysis). The higher eradication rate in the CATT group may be due to good drug compliance and avoidance of acidic food in this study.

## 5. Conclusions

In conclusion, 3-day high-frequency rabeprazole induction treatment before dual rabeprazole/amoxicillin therapy could achieve an acceptable *H. pylori* eradication rate. To achieve good treatment compliance, taking the drugs simultaneously at meal and bed times should also be recommended.

## Figures and Tables

**Table 1:** Demography and study results.

Group	HDDT	CATT	*p* Value
Patient number	36	35	
Age (y/o) ‡	59.6 ± 10.1	58.0 ± 11.0	0.13
Gender (F/M)	20/16	21/14	0.89
Smoking/alcohol	7/5	2/4	0.14
Endoscopic study			
Duodenal ulcer	8	4	0.53
Gastric ulcer	4	3	
Gastritis	27	25	
Reflux esophagitis	16	13	
Clinical presentations			
Epigastric pain	32	30	0.21
Abdomen fullness	15	16	
GERD §	22	11	
*H. pylori* eradicated success rate ‖			
Intention to treat ‖	33/36 (91.7%)	27/35 (77.1%)	0.75
95% CI	78–97%	61–87%	
Per-protocol	33/35 (94.3%)	27/32 (84.3%)	0.89
95% CI	79–99%	66–94%	

HDDT: high-dose dual therapy, rabeprazole (20 mg, four times daily for three days) induction treatment, then rabeprazole (20 mg) and amoxicillin (500 mg, four times daily for 14 days). CATT: clarithromycin, amoxicillin triple therapy, clarithromycin 500 mg, amoxicillin 1 g, rabeprazole 20 mg twice per day for 14 days. ‡ Data are presented as the mean ± standard deviation. § Gastroesophageal reflux disease. ‖ ITT, data presented as eradicated success patient number/total patient number (%). ¶ PP, data presented as eradicated success patient number/(total patients who dropped out) (%).

**Table 2:** Adverse effects in this study.

Group	HDDT (*n* =)	CATT (*n* =)	*p* Value
Taste distortion	3 (3.3%)	4 (4.0%)	0.89
Diarrhea	1(1.1%)	1 (1.0%)	0.52
Dizziness	1(1.1%)	0 (0)	0.96
Headache	2 (2.2%)	1 (1.0%)	0.94
Abdominal pain	1 (1.1%)	2 (2.0%)	0.93
Skin rash	0 (0)	2 (2.0%)	0.53
Total incidence	8 (8.9%)	10 (10.1%)	0.99

## Data Availability

The datasets used and/or analyzed during the current study are available from the corresponding author on reasonable request.
